# Uncoiling the Human Cochlea—Physical Scala Tympani Models to Study Pharmacokinetics Inside the Inner Ear

**DOI:** 10.3390/life11050373

**Published:** 2021-04-21

**Authors:** Daniel Schurzig, Max Fröhlich, Stefan Raggl, Verena Scheper, Thomas Lenarz, Thomas S. Rau

**Affiliations:** 1 MED-EL Research Center, 30625 Hannover, Germany; max.froehlich@medel.com; 2 Department of Otolaryngology, Hannover Medical School, 30625 Hannover, Germany; scheper.verena@mh-hannover.de (V.S.); lenarz.thomas@mh-hannover.de (T.L.); Rau.Thomas@mh-hannover.de (T.S.R.); 3 MED-EL Medical Electronics, 6020 Innsbruck, Austria; stefan.raggl@medel.com

**Keywords:** cochlear implantation, cochlear models, cochlear volume, drug delivery

## Abstract

In the field of cochlear implantation, artificial/physical models of the inner ear are often employed to investigate certain phenomena like the forces occurring during implant insertions. Up to now, no such models are available for the analysis of diffusion processes inside the cochlea although drug delivery is playing an increasingly important role in this field. For easy access of the cochlea along its whole profile, e.g., for sequential sampling in an experimental setting, such a model should ideally be longitudinal/uncoiled. Within this study, a set of 15 micro-CT imaging datasets of human cochleae was used to derive an average representation of the scala tympani. The spiral profile of this model was then uncoiled along different trajectories, showing that these trajectories influence both length and volume of the resulting longitudinal model. A volumetric analysis of the average spiral model was conducted to derive volume-to-length interrelations for the different trajectories, which were then used to generate two tubular, longitudinal scala tympani models with volume and length properties matching the original, spiral profile. These models can be downloaded for free and used for reproducible and comparable simulative and experimental investigations of diffusion processes within the inner ear.

## 1. Introduction

Cochlear implantation is a surgical intervention for patients with severe to profound hearing loss: in this intervention, the electrode array of a cochlear implant (CI) is inserted through the round window (or in some cases a cochleostomy) [1] into the scala tympani (ST) lumen of the cochlea [2,3]. Research aiming at the improvement of postoperative outcomes of CI surgery is often conducted experimentally, which requires the use of realistic, physical ST models. Unfortunately, the accurate representation of the ST features within a model is not trivial and requires the consideration of various factors: 

To begin with, the anatomy of the ST is highly variable [3,4,5,6]. Experimental findings derived with a model which is founded on one specific ST shape [7] may hence not hold true in general but be specific to that ST [8,9]. It may therefore be advisable to create an average model of a ST [10], but it is not clear how the known variations of the ST shape (e.g., cross-sectional profile [11] and size [3,6,12]) should be averaged without losing fundamental anatomical features of the ST [13,14]. On the other hand, certain simplifications of the ST may be tolerable if they are unlikely to influence the parameters of interest of a specific study. In terms of sufficiently accurate modelling, studies on the insertion forces occurring during electrode array insertion are most likely the most demanding investigations. The artificial cochlea models (ACM) used within these investigations must reliably feature the spiral shape of the cochlea itself [15,16,17,18], but also accurately represent the outer shape of the ST which interacts with the array during insertion [11]. Furthermore, these models should be filled with a fluid which mimics the perilymph in terms of viscosity and the frictional properties of the array sliding along the ST walls during insertion. Finally, the viscoelastic properties of intracochlear soft tissue like the basilar membrane should be included in order to cover all factors which could potentially influence insertion forces.

Drug delivery and pharmacokinetics within the inner ear are typically investigated in animal models or temporal bones [19]. However, ACMs could be employed for these types of investigations as well in order to reduce the number of animals used for experimental purposes, to diminish the need for the rarely available temporal bones and to generate reproducible and comparable results which are not blurred by anatomical variations. The requirements for these models differ substantially from those used for insertion studies since the volumetric properties of the cochlea are in the foreground. Various cell types inside the cochlea can be targeted by drug agents providing potential benefit to cochlear structures and health [20]. Furthermore, the opened round window provides the opportunity to deliver drugs directly to the ST during CI surgery [21,22,23,24], following subsequent diffusion to other regions in the inner ear [25,26]. Measuring the drug distribution along the ST in order to predict possible pharmacological effects, sequential sampling at the apex can be performed [27,28,29]. The different types of potential drug delivery and diffusion investigations highlight that models for these analyses must not only correctly represent the overall cochlear volume but the volumetric properties along the entire cochlear spiral.

The approach to create such models proposed within the present study consists of linear “uncoiled” physical ST representations to allow for easy access of the ST volume along its entire profile. The models are made available in a standardized computer aided design (CAD) format such that they can be manufactured (e.g., using 3D printing technology) for experimental drug delivery investigations. Furthermore, the CAD models could be used for simulations of diffusion processes within the inner ear (e.g., using finite element analyses) which can then be compared to the corresponding experimental findings. The main requirements for the models were to enable full insertion of drug eluting CI prototypes with no contaminants from lubricants which are needed in coiled models [30,31]. Furthermore, the perilymph volume surrounding the CI prototypes needed to be modelled accurately along the entire length of the ST, enabling the experimentalist to measure the concentration gradient along the ST by sequential sampling from base to apex.

## 2. Materials and Methods

### 2.1. Geometrical Averaging of Scala Tympani Contours

The fundamental data used for the investigation was previously presented in two earlier studies from our group [6,15]. It consists of 15 micro CT datasets in which cross sections of scala tympani (ST), scala vestibuli (SV), basilar membrane (BM) and helicotrema (HT) region were manually segmented using a custom software tool specifically designed for this task [32]. All processing of these segmentations was done in Matlab (version R2018a, MathWorks, Natick, MA, USA). 

**Figure 1:**
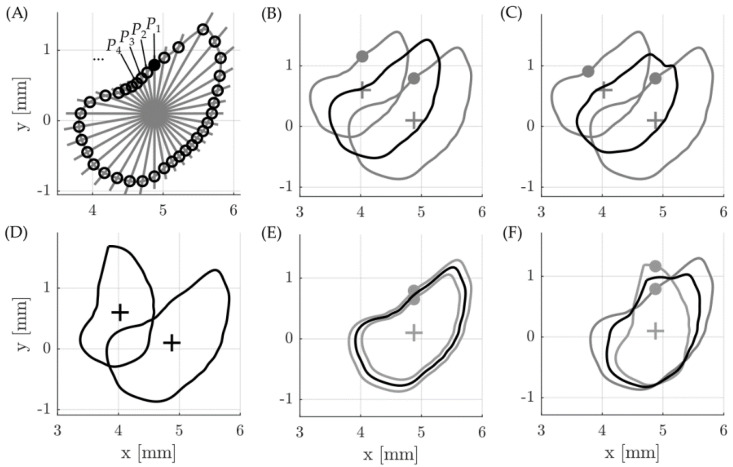
(**A**) The uniform and even distribution of contour points every 10° along the ST (scala tympani) cross section yields 36 points for each contour. The solid point P_1_ indicates the first point of each contour, which was positioned directly above each respective centroid (indicated by the cross) followed by P_2_, P_3_, P_4_ and so on in 10° intervals in counterclockwise order. This was done to (**B**) derive the correct geometrical average of different shapes and avoid (**C**) falsification of the mean contour. (**D**) Different rotational orientations of 2 cross sections (**E**) had to be taken into account prior to averaging as well in order to avoid (**F**) shape falsification when averaging.

The first step in creating an average representation of the ST was to uniformly redistribute the manual segmentation points along each cross section of each one of the individual ST segmentations. This was done by (i) interpolating the respective contour along the manual segmentation points, (ii) computing the centroid of the resulting cross-sectional area and then (iii) computing intersections of the interpolated contour and straight lines going from the centroid through the contour (Figure 1A): the first line went straight up from the centroid (yielding point P_1_, in solid), followed by 35 further lines in intervals of 10° in a counterclockwise order. While this procedure might appear quite extensive, it was found that is essential for accurate averaging of the cross-sectional geometries. Figure 1B shows an example of two cross sections of equal shape but different sizes (in gray) whose contour points are distributed according to Figure 1A. Due to the uniform point redistribution, the resulting black average profile is of the same qualitative shape as the two original profiles. Figure 1C shows the same two original shapes, but the contour points are now distributed in a slightly different manner (the solid point P_1_ of the smaller cross sections does not lie straight above its centroid). The resulting black profile does no longer have the same qualitative shape as the two original profiles.

An equally important factor to be considered is the rotation of the cross sections [6]. Figure 1D shows two qualitatively identical cross sections of different size and with a different angular orientation. If the cross sections are rotated to the same orientation, the resulting average shape is qualitatively identical to the original ones (Figure 1E). In contrast, thereto, neglecting the different angular orientations yields a shape falsification when averaging. That is why all shapes were rotated to the cochlear angle dependent average orientation of the BM [6] prior to point redistribution and averaging.

The described methodology consisting of (1) equal point distribution, (2) the consideration of individual, cross-sectional rotations, (3) averaging of contour point coordinates and (4) rotating the average shape back to the average rotation at the respective angular location, was used to average the cross sections of all 15 ST segmentations.

### 2.2. CAD Model Generation and Analysis

**Figure 2:**
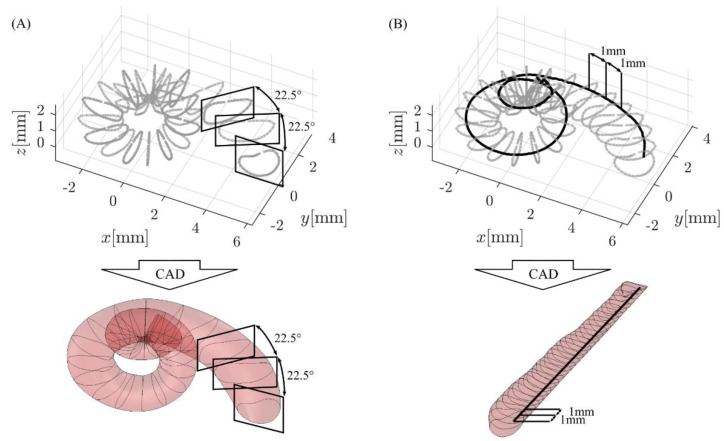
CAD (computer-aided design) model generation in SolidWorks: (**A**) spiral models were generated with the *loft* feature using cross-sectional information in 22.5° steps, whereas (**B**) straight models uncoiled along a specific trajectory (solid black line) were created based on cross-sectional information in 1 mm intervals along this trajectory.

The fundamental methodology to create volumetric models out of the averaged contour points was previously proposed in [32]: using Matlab, the points were exported in a format which can be read by a CAD software. In case of this study, the *sldcrv* format was used such that the contour points could be imported into SolidWorks (version 17.05.0089, Dassault Systèmes, Vélizy-Villacoublay, France). The contour points were then imported into SolidWorks and connected to create a solid body using the *loft* feature, yielding the CAD mean model of the ST shown in Figure 2A.

Uncoiled models of the ST along a specific spiral path were created as follows (see Figure 2B): in Matlab, the spiral profile of interest of the average ST was divided into 1 mm segments, and the ST cross-section for each 1 mm step was computed. These cross-sections were then extracted from the ST model and rotated/uncoiled such that they lie on the *xy* plane with the intersection point of spiral and cross-section on the *z* axis. Each cross section was then shifted along the *z* axis according to the respective length value along the spiral (e.g., the cross section of the 5th 1 mm segment was shifted 5 mm along *z*). These reoriented cross-sections were then exported as before and connected in SolidWorks using the *loft* feature (Figure 2B). Within this study, the spiral profiles were chosen such that they correspond to an uncoiling of the ST along the ST lateral wall (LW) as well as 0.35 mm and 0.5 mm off the LW, which are assumed to correspond to the insertion path of straight CI electrode arrays (i) and the location of the Organ of Corti (OC), respectively [33,34,35].

After generating the different CAD models, volume information of the respective models was determined using the *mass properties* tab in SolidWorks. If volume information of specific sections of a model needed to be computed, the model was cut using the *cut with surface* feature to separate the section of interest and all other section were then (temporarily) deleted using the *delete/keep body* feature.

## 3. Results

**Figure 3:**
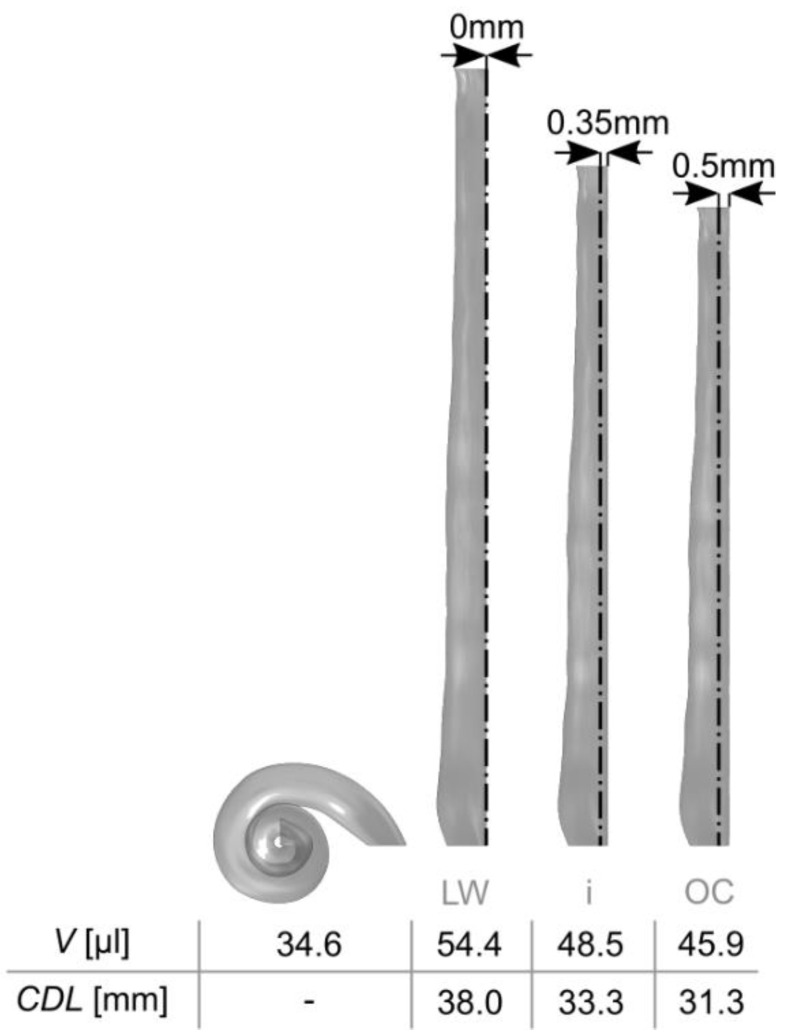
Uncoiling the mean model of the ST along different paths along the spiral affects both the ST volume and length of the uncoiled model. V: volume; CDL: cochlear duct length; LW: lateral wall; i: insertion path of straight CI electrode array; OC: Organ of Corti.

The derived CAD models of the average ST in its original, spiral representation as well as uncoiled along the LW, CI insertion trajectory and OC respectively are depicted in Figure 3. The depiction also includes information on the respective lengths and volumes. It is shown that the spiral ST structure uncoiled along the LW yields a model of 38.0 mm length with a volume that is 57% larger than the original, spiral representation. Uncoiling the ST along the CI insertion and OC paths (i.e., with an offset of 0.35 mm and 0.5 mm off the LW respectively [33]) causes a reduction of both model length and volume comparted to the LW trajectory; however, the volume is still 40% too large for the insertion and 33% too large for the OC paths, respectively. It is hence not trivial to create a straight representation of the cochlea which accurately mirrors the volumetric surroundings of a straight electrode array or the OC inside the ST.

**Figure 4:**
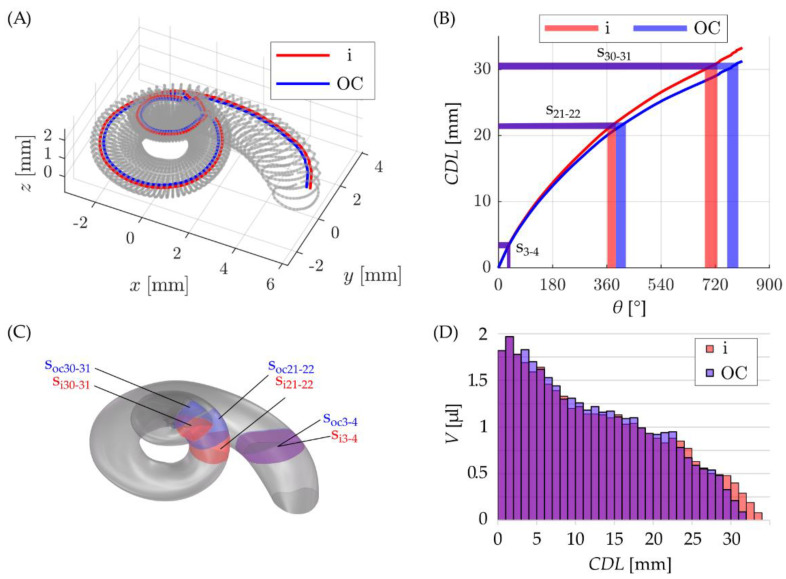
(**A**) visualization of insertion (in red) and OC (in blue) paths inside the ST, (**B**) the length of these paths along the cochlear spiral including an exemplary visualization of three cochlear sections corresponding to CDL intervals of 3–4 mm (s_3–4_), 21–22 mm (s_21–22_) and 30–31 mm (s_30–31_) along the insertion and OC trajectories, respectively. (**C**) Visualization of these cochlear sections in the CAD model and (**D**) a depiction of all sectional volumes along the insertion and OC paths.

In order to create uncoiled ST models with an accurate volumetric representation of the spiral model, two straight ACMs were developed in the following manner: as before, the distances of 0.35 mm and 0.5 mm from the LW were used to approximate the location of a straight CI array (i) and OC along the ST, respectively (Figure 4A). The lengths CDL_i_ and CDL_OC_ of these trajectories along the angle were then computed and divided into sections of 1 mm length and the angular values corresponding to these intervals were noted.

The resulting curves *CDL*_i_(θ) and *CDL*_OC_(θ) as well as three examples of 1 mm sections along the insertion paths are depicted in Figure 4B (s_3__–4_: 29° ≤ *θ*_i_ ≤ 40°, 29° ≤ *θ*_OC_ ≤ 41°; s_21__–22_: 362° ≤ *θ*_i_ ≤ 390°, 390° ≤ *θ*_OC_ ≤ 423°; s_30__–31_: 686° ≤ *θ*_i_ ≤ 727°, 760° ≤ *θ*_OC_ ≤ 796°).

The derived angular values of the 1 mm sections were then used to cut out the corresponding radial sections of the spiral ST CAD model (Figure 4C) and note the respective section volumes computed in SolidWorks (Figure 4D, e.g., s_3–4_: *V*_i_ = 1.69 µL, *V*_OC_ = 1.83 µL; s_21–22_: *V*_i_ = 0.83 µL, *V*_OC_ = 0.94 µL; s_30–31_: *V*_i_ = 0.40 µL, *V*_OC_ = 0.21 µL).

This derived information on path lengths and ST volumes depicted in Figure 4D was then used to compute straight ST models which match the spiral ST shape in both length and volume. This was done by computing truncated cones of 1 mm height for each 1 mm segment of each path such that the volume of each cone matches the corresponding volume computed in SolidWorks: for the section s_i3–4_, for instance, the volume of the truncated cone is computed as (Equation (1))

*V*_i3–4_ = 1/3∙π∙(r_i3_^2^ + r_i3_r_i4_ + r_i4_^2^)∙1 mm. (1)

**Figure 5:**
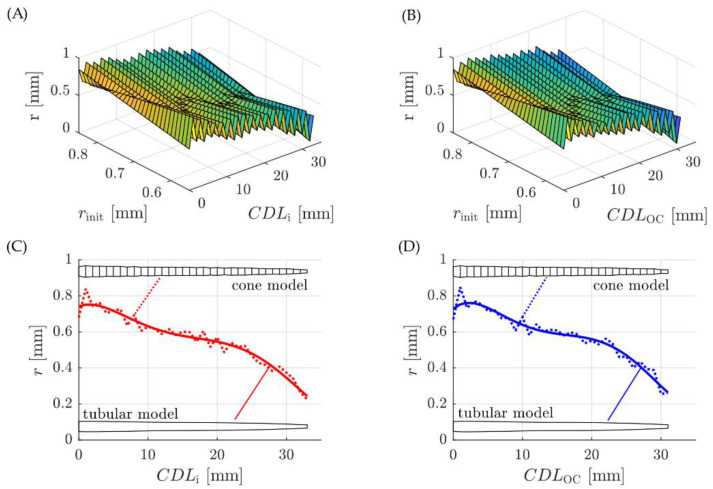
Cone radii r for different initial radii r_init_ along the (**A**) insertion and (**B**) OC (organ of Corti) path length (*CDL*). Depictions of the cone models with minimal change in radii and the fitted tubular models for the volumetric (**C**) insertion and (**D**) OC representations.

Since each radius *r* affects the volume of the two neighboring volumes (i.e., radius r_i3_ plays into *V*_i2–3_ and *V*_i3–4_), the choice of the initial radius r_init_ at *CDL* = 0 mm defines the entire profile of the cone model. Figure 5A, B show how the cone radii r along the respective *CDL*s are dependent on the initial radius r_init_: if the latter is chosen too high or low, the cone radii along the model become very inconsistent. In order to find the smoothest profile possible, the change in cone radii for each one of the initial radii from r_init_ = 0.5 mm to r_init_ = 1.0 mm (in 0.01 mm steps) along the CDL was evaluated according to (Equation (2))

dr *= ∑*(r_k_ − r_k−1_)^2^ (2)

from k = 1 to k = n (where *n* denotes the total number of cone radii and hence, total length of the model). The smallest changes in cone radii were found for r_init_ = 0.68 mm for the insertion model and r_init_ = 0.67 mm for the OC model, respectively. The corresponding cones models are depicted in Figure 5C, D and although the cone models are relatively smooth the change in radii is still noticeable (dashed lines). That is why a fifth order polynomial function was fitted to these radii, yielding the corresponding tubular models without any sudden, unnatural changes in model diameter (solid lines).

**Figure 6:**
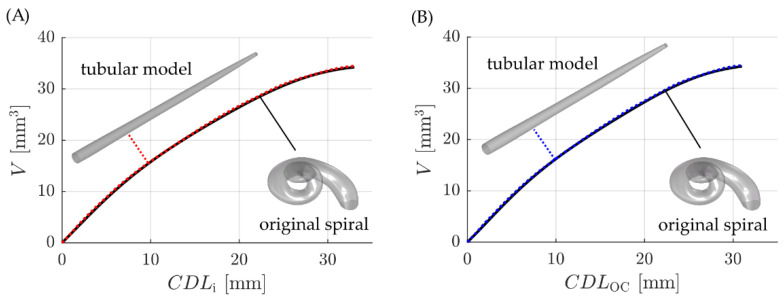
Comparison of the volume-to-length ratios for the original ST spirals (black) and the corresponding tubular representations for (**A**) the insertion (red) and (**B**) OC (blue) trajectories respectively.

Finally, it was verified that these tubular models for the i and OC trajectories accurately represent the volume-to-length ratios originally computed for the spiral ST mean model (i.e., Figure 4D). In order to do so, the volume along the i and OC trajectories of the spiral model was compared to the longitudinal volume of the corresponding tubular models. The corresponding results are depicted in Figure 6 and show a very good agreement of the reference profiles and model representations.

## 4. Discussion

Pharmacotherapy is a hot topic in cochlear implant optimization. Next to the development of technical aspects [36,37], the drug-mediated modulation of the biological processes in the inner ear is of crucial importance. Technological and biological strategies are therefore no longer decoupled. Instead, technical improvements could result in a much larger benefit for the patient if due to the additional pharmacotherapy, the cochlear neural structures are protected, the dendrites of the spiral ganglion cells are regenerated and stimulated to grow out towards the electrode, the post-operative fibrosis around the implant is reduced and residual hearing is preserved [38]. Pharmacologically addressing those biological targets influences the implantation outcome for the patient. Hence, estimates for the drugs to be delivered should be as precise as possible to create improved and more reliable clinical outcomes. The ultimate/long term goal is to develop a method for calculating which amount of which drug has to be incorporated in a specific delivery matrix to achieve the expected biological response in the individual patient.

The described approach is a further step toward the development of standardized, model-based investigations of drug delivery strategies for local inner ear therapy. Using the presented models, these studies can be conducted using simulation environments and/or experimental settings: physical prototypes of the derived models (e.g., via 3D printing) allow for sequential, longitudinal sampling of the drug concentration in a reliable manner due to the accurate volumetric representation of the ST (compare e.g., with [39]). Fluid sampling can be conducted without the restraints of coiled cochlear models since the volume of the ST is easily accessible along the entire length of the ST. Based on these studies, drug related parameters such as molecule size, matrix material, the drug release rate as well as the stability of matrix and drug [28] can be investigated in detail. The uncoiled models are freely available for download and use under https://vianna.de/acms.html (accessed on 21 April 2021).

The different steps necessary to develop this uncoiled representation of an average ST demonstrated that the generation of such a model is not trivial. Even the averaging of different ST cross-sections, i.e., the foundation of creating uncoiled ST mean models, is rather complex: recent studies demonstrated that not only differences in size [3] but also the orientation of cochlear cross-sections is variable in between specimens [6]. Simple averaging without the consideration of this orientation [7] is hence less likely to preserve common anatomical features of the cross-sectional geometry. Furthermore, simple uncoiling of the spiral ST geometry affects both length and volume of the resulting model such that comparability to the original, spiral representation is no longer given (cf. Figure 3), an effect also observed by Salt et al. [40].

The two models developed in this study represent the uncoiling of the ST along the spiral profiles of the insertion trajectory of straight electrode arrays as well as the OC, which are assumed to lie approximately 0.35 mm and 0.5 mm away from the LW toward the modiolus respectively [33]. The difference between these two paths is hence merely a radial offset of 0.15 mm and the overall, qualitative profile of the resulting uncoiled models is very similar (Figure 6). However, differences are visible especially as one moves further toward the apex (Figure 4D): in this region, the cochlea is wrapped more tightly such that the relative effect of the 0.15 mm difference becomes more noticeable. Furthermore, despite the small radial difference of the insertion and OC trajectories respectively, the length of the corresponding spiral paths (Figure 4) and hence the uncoiled models (Figure 6) show a difference of 2 mm. It should also be noted here that the consistent offsets of 0.35 mm and 0.5 mm are simplified assumptions regarding the location of straight electrode arrays and the OC respectively. Both straight arrays and the OC were found to have different distances to the LW at different locations along the cochlear spiral [41,42]. The derived models are hence volumetrically accurate models of the uncoiled ST, however, they are only approximations of volumetric representations of the ST along straight arrays and the OC respectively.

The derived, uncoiled mean models of the ST are an important step toward repeatable and comparable investigations of diffusion processes within the human inner ear [43]. In contrast to previously proposed models, they are founded on an average model of 15 different human cochleae and were uncoiled with the premises of preserving the volumetric properties of the cochlea (compare e.g., with [40,44,45]). The models were generated such that they can be employed for both simulations and experiments on drug delivery processes within the inner ear, which allows for the comparison of the respective results. However, the derived mean model is still a model and lacks important properties of harvested cochleae like the perilymph and soft tissue inside the ST. Furthermore, the impact of anatomical variations in between specimens would require the development of several of these models with the methods described. However, the uncoiled models could potentially serve as a suitable replacement for harvested cochleae in terms of fundamental studies on novel drugs or processes to apply these drugs.

## 5. Conclusions

A method for volumetrically accurate modeling of the uncoiled, mean scala tympani anatomy has been developed and validated within this study. Two artificial cochlear models were derived using this method, and the uncoiled shapes of these models substantially simplify the investigation of diffusion processes within the inner ear, allow for repeatable and comparable analyses in both simulative and experimental environments and could hence serve as a replacement for harvested animal cochleae.

## Data Availability

The derived models of the uncoiled scala tympani can be downloaded under https://vianna.de/acms.html (accessed on 21 April 2021).
